# Vida PURA: results from a pilot test of culturally adapted screening and brief intervention for Latino day laborers

**DOI:** 10.1186/1940-0640-10-S2-O26

**Published:** 2015-09-24

**Authors:** India Ornelas, Bonnie Duran, Dennis Donovan

**Affiliations:** 1 Health Services, University of Washington, Seattle, USA; 2 Social Work, University of Washington, Seattle, USA; 3 Psychiatry and Behavioral Sciences, University of Washington, Seattle, WA

## Background

The aim of this study was to assess the feasibility of culturally adapted screening and brief intervention to reduce heavy alcohol use among Latino day laborers.

## Material and methods

We conducted qualitative interviews with Latino day laborers and social service to inform the cultural adaptation of screening and brief interventions. Case summaries and coded transcripts were reviewed for prevalent themes. Themes were used to identify sources of mismatch between traditional screening and brief intervention and the target population. The adapted intervention was then pilot tested to assess the feasibility and potential effectiveness. In the pilot test, 104 men were screened using the AUDIT and men with a score 6 or greater were offered a brief intervention (56%). Those receiving an intervention completed follow-up surveys at 2 and 8 eight weeks. Alcohol use was assessed using the AUDIT and 14 day timeline follow-back.

## Results

Findings from the qualitative interviews indicated that unhealthy drinking was related to and helped relieve immigration-related stressors. They faced many barriers to accessing health and social services and few culturally-appropriate alcohol-related services existed. Based on these findings, we adapted SBI to incorporate the social and cultural context of Latino day laborers. SBI was provided in a community setting (at a day labor worker center) by bilingual community health workers. Men were receptive to SBI during the pilot test. Results from the pilot test confirmed that unhealthy alcohol use was prevalent (average of 8.5 drinks per drinking day and 4 drinking days in past 14 weeks among intervention group). Mean AUDIT scores among those receiving the intervention went from 18.7 at baseline, to 13.5 at 2 weeks, and 14.8 at 8 weeks.

## Conclusions

Our results suggest that screening and brief intervention may be more efficacious for Latino day laborers if conducted by community health workers in community settings. Our findings can be used to further test culturally adapted SBI to prevent and reduce unhealthy alcohol use in this vulnerable population.

